# Evaluation of the Quilting Technique for Reduction of Postmastectomy Seroma: A Randomized Controlled Study

**DOI:** 10.1155/2015/287398

**Published:** 2015-07-12

**Authors:** Ashraf Khater, Waleed Elnahas, Sameh Roshdy, Omar Farouk, Ahmed Senbel, Adel Fathi, EmadEldeen Hamed, Mohamed Abdelkhalek, Hosam Ghazy

**Affiliations:** ^1^Department of Surgical Oncology, Mansoura Oncology Center (OCMU), Faculty of Medicine, Mansoura University, Dakahlia, Egypt; ^2^General Surgery Department, Mansoura University Hospital, Mansoura University, Dakahlia, Egypt

## Abstract

*Background*. Postmastectomy seroma causes patients' discomfort, delays starting the adjuvant therapy, and may increase the possibility of surgical site infection. *Objective*. To evaluate quilting of the mastectomy flaps with obliteration of the axillary space in reducing postmastectomy seroma. *Methods*. A randomized controlled study was carried out among 120 females who were candidates for mastectomy and axillary clearance. The intervention group (*N* = 60) with quilting and the control group without quilting. All patients were followed up routinely for immediate and late complications. *Results*. There were no significant differences between the two groups as regards the demographic characteristics, postoperative pathological finding, and the immediate postoperative complications. The incidence of seroma was significantly lower in the intervention group compared with the control group (20% versus 78.3%, *P* < 0.001). Additionally, the intervention group had a shorter duration till seroma resolution (9 days versus 11 days, *P* < 0.001) and a smaller volume of drainage (710 mL versus 1160 mL, *P* < 0.001) compared with the control group. *Conclusion*. The use of mastectomy with quilting of flaps and obliteration of the axillary space is an efficient method to significantly reduce the postoperative seroma in addition to significantly reducing the duration and volume of wound drainage. Therefore we recommend quilting of flaps as a routine step at the end of any mastectomy.

## 1. Introduction

Seroma is one of the most bothersome events that disturbs both the patient and surgeon with multiple visits that delay starting the adjuvant therapy and cause great patients' discomfort with a possibility of increased surgical site infection [1–3]. Postmastectomy seroma can be defined as a collection of serous fluid just under the skin flaps or in the axillary pace immediately following mastectomy with axillary dissection that can be detected either clinically or sonographically [4, 5]. Seroma is graded 1 if asymptomatic (only diagnosed by ultrasound), graded 2 if symptomatic but can be managed either medically or by simple aspiration, and graded 3 if symptomatic and requires surgical or radiologic intervention [1]. The incidence of seroma following mastectomy and axillary clearance varies in reports from 25% to 60% [6], with even higher incidences being reported [1]. Despite the extensive investigation [7–15], the exact pathogenesis of postmastectomy seroma is still not fully understood. However a significant correlation was discovered with the volume of drainage in the first three postoperative days [8, 16], especially when exceeding 500 mL [17]. In a controlled randomized study that was carried out by Lumachi et al. in 2004, the total amount of drainage was independently correlated with seroma formation [18]. In this important study the use of the ultrasonic dissector significantly reduced seroma formation [18]. On the other hand, in another important controlled randomized study that was done by Porter et al. in 1998, the use of electrocautery was significantly associated with increased seroma formation when compared with flap elevation and dissection of the fascia by the scalpel [19]. Other implicated factors have been described in the evidence based search by Kuroi et al. and a systematic review by van Bemmel et al. [15, 20]. The first research group to report the concept of flap fixation to significantly reduce the development of seroma was Chilson et al. in 1992 [13].  Table 1 shows summary of studies that utilized this technique with a significant reduction in the incidence of seroma [3, 5, 13, 21–23].

The aim of the current study was to evaluate the efficacy of mastectomy with quilting of flaps and obliteration of the axillary space in reducing postmastectomy seroma.

## 2. Patients and Methods

### 2.1. Patients

A planned number of 120 operable female patients who were candidates for total mastectomy and axillary clearance were enrolled in this study. Patients with inoperable disease, those who received prior chemotherapy or breast irradiation, those with prior breast surgery, those with morbid obesity, those with collagen disease, those with poorly controlled diabetes, and those with history of long term use of steroids were excluded from this study.

### 2.2. Design

A randomized controlled study was carried out in the period from February 2012 to September 2014. Written consent was obtained from all patients prior to enrollment. After obtaining all required ethical approvals from ethical committees at the Mansoura University Hospital and Mansoura Oncology center (OCMU), patients were randomized into equal two groups using a computer generated random number. In the first (intervention) group (*n* = 60), mastectomy was done using the scalpel with cautery of the bleeding points only with quilting suture of both the upper and lower flaps to the underlying pectoral fascia together with obliteration of the axillary space as well while, in the second (control) group (*n* = 60), mastectomy was done in the same way without quilting.

### 2.3. Intervention

**Figure 1:**
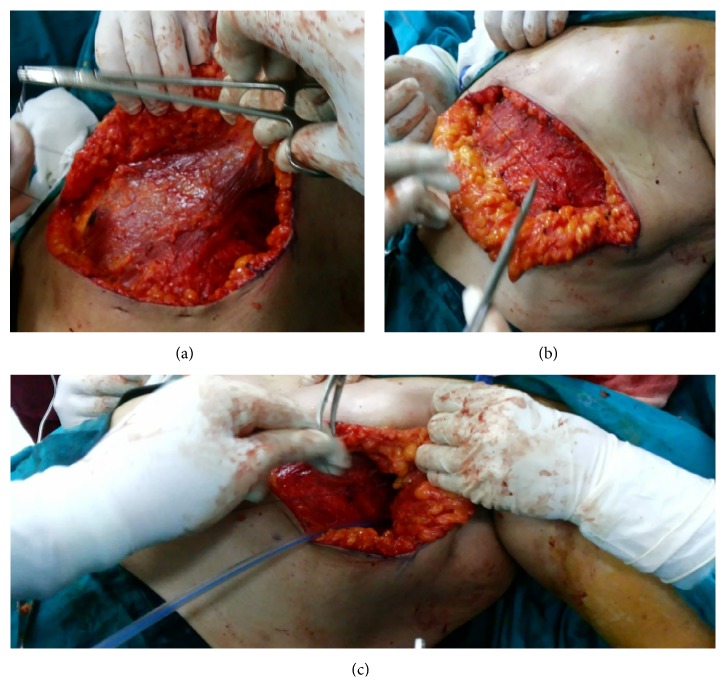
Quilting of the upper flap (a), the lower flap (b), and the axilla (c).

Using Vicryl 2/0 suture, we started the quilting technique in the upper flap from medial to lateral by a continuous suture that fixes the undersurface of the upper flap to the pectoral fascia with care to avoid entangling the dermis which results in unsightly dimpling. The second row was done by the same continuous suture from lateral to medial till the medial angle. The same was done for the lower flap. Lastly the axilla was obliterated by suturing its lateral wall to the fascia of the serratus anterior and medial axillary wall (Figure 1). An 18 French tube drain was inserted in the axilla in all cases of the study.

### 2.4. Patients Follow-Up

All patients were followed up routinely for immediate and late complications including hemorrhage, flap necrosis, and wound sepsis. Patients were typically discharged at the second postoperative day with instructions for home drain care, assisted by regular visits for recording of the total drainage volume before drain removal, the amount of drainage fluid in the first 3 days, the amount of drainage in the last 3 days before removal, and the duration till drain removal (drain was removed when the 24-hour effluent was less than 50 mL). Seroma was recorded when detected either clinically or sonographically (on routine postoperative ultrasound evaluation). If seroma was diagnosed, we recorded the number of aspirations till resolution, the total aspirated volume, and the number of days before complete resolution. The patients' pathological data was also recorded.

### 2.5. Statistical Analysis

Data were presented as frequencies and percentages for categorical data and mean, standard deviation (SD), and range for continuous data. The association between categorical variables was examined using Chi Square Test (*χ* ^2^). The difference in mean values of continuous data was examined using independent-samples *t*-test. All *P* values were two-tailed. *P* value <0.05 was considered as significant. SPSS software (release 15.0, SPSS Inc., Chicago, US) was used for statistical analyses.

## 3. Results

The mean age of the quilting (study) group was 46 ± 7 years (range 38–65), while in the control group it was 44 ± 8 with (range 37–66), with a nonsignificant difference (*P* value  =  0.14). Similarly, no significant differences were detected between the two study groups as regards the baseline body mass index, tumor size, pathologic type, and pathologic grade (Table 2).

The operative outcome among the two study groups is shown in (Table 3). The operative time was prolonged in the quilting group by around 20 minutes (*P* < 0.001). The number of positive lymph nodes was nearly similar in both groups. The duration before drain removal was shorter in the intervention group (9 days, range 7–20) than the control group (11 days, range 9–18), and the difference was highly significant (*P* < 0.001). Similarly, compared to the control group, the intervention groups had smaller total drainage volume (710 mL versus 1160 mL, *P* < 0.001), smaller amount of effluent in the first three days (230 mL versus 425 mL, *P* < 0.001), and smaller output in the last three days (180 mL versus 231 mL, *P* = 0.02). Additionally, the average number of aspirations till disappearance of the seroma and the average number of days till seroma disappearance were smaller in the intervention group compared with the control groups (2.1 versus 4.7 aspirations and 2.3 versus 10 days, resp., *P* < 0.001 for each).

There were no significant differences between the two groups as regards the postoperative hematoma and flap necrosis (Table 3). The incidence of seroma in the intervention group was 20% (12/60 patients) compared to 78.3% (47/60 patients) in the control group, and the difference was highly significant (*P* < 0.001).

## 4. Discussion

We are reporting the efficacy of the use of mastectomy with quilting of flaps and obliteration of the axillary space in reducing the postmastectomy seroma using a randomized design. Several trials used adhesive glues and sclerosant agents to reduce the postmastectomy seroma. However, a recent meta-analysis showed that such preventive techniques are still not convincing [20]. As electrocautery was associated with a higher incidence of seroma formation, there was a trend towards the use of scalpel mastectomy [19] or harmonic shear [24].

The concept of suturing the skin flaps to the underlying muscle and obliteration of the axillary space is not new. Actually, closure of the dead space especially in the axilla was recommended by van Bemmel et al. [20].  Table 1 shows most of the trials that used fixation of the skin flaps to the underlying muscles and fascia. However most of these studies were nonrandomized [13, 21–23]. In our study the incidence of seroma in the quilting group was 20% versus 78.6% in the control group (Table 3). This was in line with that described by Coveney EC and his coworkers in 1993 who described an incidence of 25% in the suture group versus 85% in the control group [21]. A similar figure was reported by Sakkary MA in 2012 in a small study (20 patients per each arm) with an overall incidence of 20% in the intervention group versus 50% in the control group (*P* = 0.047) [5]. In a more recent study by Ten Wolde and his coworkers in 2014, there was a decrease of seroma from 80.5% in the control group to 22.5% in the quilting group (*P* < 0.01) [3].

The quilting maneuver in the current study had significantly decreased the total drainage volume (from a mean of 1160 to a mean of 710) (Table 3). Similar results were reported by Sakkary with a mean decrease from 2017.8 mL in the control group to 524.8 in the intervention group (*P* < 0.001) [5]. Additionally, the quilting in the current study significantly reduced the mean duration of drainage from 11 days in the control group to 9 in the intervention group (*P* < 0.001). A similarly significant finding was described by Sakkary (from 13.4 to 5, *P* < 0.001). The quilting in the current study was found to significantly decrease the mean days to seroma disappearance, the total aspirated volume, and the number of aspirations (Table 3). This was similar to that reported by Ten Wolde and his coworkers, which is a decrease of mean number of aspirations from 4.86 to 2.40 (*P* = 0.015) and the volume of aspirations from 1660 mL to 611 mL (*P* = 0.05). There was a significant correlation between the extent of mastectomy and seroma formation being more significant with modified radical mastectomy than with breast conserving surgery [10, 11]. It was not a surprise to find that this maneuver prolongs the operative time significantly (Table 3) and we consider this the expense for reduction of postmastectomy seroma and all its sequels. Similar to our findings, neither the number of dissected axillary lymph nodes nor the extent of dissection influenced seroma formation significantly [12, 13]. However Purushotham et al. demonstrated in a controlled randomized study that the incidence of seroma was significantly lower with sentinel lymph node (SLNB) axillary approach than with conventional axillary dissection [14]. Neither the number of drains nor the nature of the drain (suction or passive) significantly influenced the seroma formation [15].

## 5. Conclusion

The use of mastectomy with quilting of flaps and obliteration of the axillary space is an efficient method to significantly reduce the postoperative seroma in addition to significantly reducing the duration and volume of wound drainage. Therefore we recommend quilting of flaps as a routine step at the end of any mastectomy.

## Figures and Tables

**Table 1:** Studies that investigated closure of the dead space after mastectomy.

Study/year	Type of trial	Number	Intervention	Result
Chilson et al. 1992 [13]	Retrospective Level 3	351	MRM with or without suture flap fixation	Suture flap fixation Sig. ↓ seroma

Coveney et al. 1993 [21]	RCT Level 2	39	Suture flap fixation versus conventional skin closure	25% incidence of seroma versus 85% (*P* < 0.001)

Purushotham et al. 2002 [22]	RCT Level 2	375	Mastectomy, BCS No drainage with suture flap fixation versus drainage without suture flap fixation	61% versus 55% (NS) in MRM, 47% versus 51% (NS) in BCS

Schuijtvlot et al. 2002 [23]	Prospective Level 2	97	BCS without drainage suture flap fixation (buttress suture) without drainage versus conventional surgery	Suture flap fixation Sig. ↓ seroma

Sakkary 2012 [5]	Prospective	40	MRM with or without suture flap fixation	Suture flap fixation Sig. ↓ seroma

Ten Wolde et al. 2014 [3]	Retrospective	176	MRM with or without suture flap fixation	Suture flap fixation Sig. ↓ seroma

**Table 2:** Demographic and clinical data of the two study groups^*∗*^.

	Intervention group	Control group	Statistics^*∗∗*^	*P* value
	(*n* = 60)	(*n* = 60)		
Age (years)	46 ± 7	44 ± 8	1.5	0.14
	[38–65]	[37–66]		

BMI	30.5 ± 1.8	30.9 ± 1.5	1.3	0.19
	[29–35]	[27–34]		

Tumor size (mm)	35 ± 6	34 ± 7	0.8	0.4
	[24–65]	[25–66]		

Pathologic Type	46 (76.7%)	47 (78.3%)	0.0	1.00
(Ductal versus others)				

Grade (Grade 1 versus 2 and 3)	8 (13.3%)	11 (18.3%)	0.25	0.62

^*∗*^Data were presented as mean ± SD [range] for continuous data and number (%) for categorical data.

^*∗∗*^ *t*-test for continuous data and Chi square for categorical data.

**Table 3:** Operative data and outcome among the two study groups^*∗*^.

	Intervention group (*n* = 60) mean ± SD [Range]	Control group (*n* = 60) Mean ± SD [Range]	Statistics^*∗∗*^	*P* value
Operative time in minutes	127 (10.5) [90–160]	105 (7.5) [80–139]	9	<0.001

The total number of lymph nodes	19 ± 3 [13–24]	18 ± 3 [12–23]	1.8	0.07

The number of positive lymph nodes	3.3 ± 1.5 [0–9]	3.6 ± 1.6 [0–10]	1.1	0.30

Duration before drain removal (days)	9 ± 3 [7–20]	11 ± 3 [9–18]	3.7	<0.001

Total drainage volume (mL)	710 ± 290 [300–1500]	1160 ± 420 [500–2000]	6.8	<0.001

Volume in the first 3 days (mL)	230 ± 112 [120–550]	425 ± 143 [130–650]	8.3	<0.001

Volume in the last 3 days (mL)	180 ± 88 [130–350]	231 ± 134 [150–450]	2.5	0.02

Seroma	12 (20%)	47 (78.3%)	38.5	<0.001

Days till seroma disappearance	2.3 ± 4.9 [0–12]	10 ± 4.2 [0–12]	9.2	<0.001

Numbers of aspirations	2.1 ± 0.6 [2–4]	4.7 ± 2.1 [3–7]	9.22	<0.001

Total volume of aspirations (mL)	45 ± 15 [0–250]	189 ± 60 [0–380]	18	<0.001

Postoperative hematoma	18 (30%)	16 (26.7%)	0.04	0.80

Flap necrosis	5 (8.3%)	6 (10%)	0.0	1.00

^*∗*^Data were presented as mean ± SD [range] for continuous data and number (%) for categorical data.

^*∗∗*^ *t*-test for continuous data and Chi square for categorical data.
